# LncRNA Expression Profiling of Ischemic Stroke During the Transition From the Acute to Subacute Stage

**DOI:** 10.3389/fneur.2019.00036

**Published:** 2019-02-01

**Authors:** Wenli Zhu, Lili Tian, Xuanye Yue, Jingyi Liu, Ying Fu, Yaping Yan

**Affiliations:** ^1^Department of Neurology, Tianjin Neurological Institute, Tianjin Medical University General Hospital, Tianjin, China; ^2^Key Laboratory of the Ministry of Education for Medicinal Resources and Natural Pharmaceutical Chemistry, National Engineering Laboratory for Resource Development of Endangered Crude Drugs in Northwest of China, College of Life Sciences, Shaanxi Normal University, Xi'an, China; ^3^Department of Biochemistry, Smith College, Northampton, MA, United States

**Keywords:** ischemic stroke, lncRNA profiling, mRNA profiling, peripheral immune reaction, RNA-seq

## Abstract

Ischemic stroke induces profound effects on the peripheral immune system, which may participate the infectious complications. However, the exact function and mechanism of immune reaction in stroke development are not well-elucidated. Recently, several long non-coding RNAs (LncRNAs) are reported to affect ischemic stroke process, especially the immunological response after stroke. In the present study, we investigated the profile of LncRNAs in human ischemic stroke during the transition from the acute to subacute stage, when the state of the peripheral immune system changes from activation to systemic immunosuppression. In this study, we analyzed the RNA-sequencing (RNA-seq) datasets obtained at two time points (24 h and 7 days) from the peripheral blood mononuclear cells of ischemic patients. Vascular risk factor-matched healthy adults were enrolled as controls. A total of 3,009 LncRNAs and 3,982 mRNAs were identified as differentially expressed 24 h after stroke. Furthermore, 2,034 LncRNAs and 1,641 mRNAs were detected to be differentially expressed on day 7. Bioinformatics analyses, including GO, KEGG pathway enrichment analysis, and network analysis, were performed for the identified dysregulated genes. Our study reveals that ischemic stroke can influence the expression of LncRNAs and mRNAs in the peripheral blood at both the acute and subacute stages; the level of LncRNAs in the antigen processing and presentation pathway was clearly upregulated at 24 h and had recovered to normal levels on day 7 after stroke. Moreover, inflammatory mediator regulation of TRP channels and GABAergic synapses were two specifically downregulated pathways on day 7 after stroke. Our findings provide a valuable resource for further study of the role of LncRNAs in peripheral immune system changes following ischemic stroke.

## Introduction

Acute ischemic stroke (IS) is a major cause of mortality and disability worldwide, and the incidence is increasing (1). Despite advances in the cause and acute treatment of ischemic stroke, its exact pathophysiologic mechanisms are still not completely understood. However, accumulating evidence has shown that long non-coding RNAs (LncRNAs) play critical roles in the pathogenesis of rodent (2, 3) and human ischemic stroke (4).

LncRNAs, which are defined as transcripts of more than 200 nucleotides that are not translated into proteins, have historically been regarded as junk DNA. However, with the development of high-throughput technologies, recent studies have revealed that many non-coding RNAs play critical roles in cellular homeostasis (5, 6). The functions of LncRNAs have been proposed to include organization of nuclear domains, cis-, or transregulation of transcription, regulation of proteins or RNA molecules, and even encoding of small proteins (7–9). Moreover, several LncRNAs have been shown to play roles in animal models of stroke and oxygen-glucose deprivation (OGD) cell models (2, 10). Using genome-wide RNA-seq, Bhattarai et al. found that transient focal ischemia induces widespread changes in the expression of LncRNAs in the mouse cortex, and a plethora of those LncRNAs were unannotated (11). After exposure to OGD, which mimics ischemic conditions *in vitro*, the expression of LncRNAs changed significantly in mouse brain microvascular endothelial cells (12). As there is a species difference between rodents and humans, the lncRNA profile after ischemic stroke in patients definitely needs to be investigated. However, recent studies on the issue focused only on a limited number of LncRNAs, such as H19 (10) and antisense non-coding RNA in the INK4 locus (ANRIL) (13). While a large number of LncRNAs remain undiscovered in human ischemic stroke, the functions of most LncRNAs have not yet been elucidated (6, 14). Thus, we used next-generation sequencing technology to characterize the systemic gene expression of peripheral blood mononuclear cells (PBMCs) in ischemic stroke patients.

In addition, ischemic stroke induces profound effects on the peripheral immune system, the state of which changes from activation to systemic immunosuppression during the transition from the acute to subacute stage (15). However, why and how the peripheral immune system participates in stroke pathology and whether LncRNAs take part in stroke pathology are largely unknown. To address this gap in knowledge, in this work, we used unbiased high-throughput RNA-seq to determine the genome-wide expression of LncRNAs in PBMCs from patients following acute ischemic stroke and investigated changes in lncRNA expression over the acute to subacute transition phase of ischemic stroke.

## Materials and Methods

### Study Subjects

Acute ischemic stroke patients and control subjects were recruited between 2015 and 2016 from Tianjin Medical University General Hospital, China. The present study was approved by the Ethics Committee of Tianjin Medical University General Hospital, and written informed consent was provided by the participants or their proxy. We enrolled 5 patients (10 samples) between the ages of 50 and 75 years with first-ever acute ischemic stroke defined as acute lesion on diffusion-weighted image (DWI), corresponding acute neurological deficit and anterior or middle cerebral artery occlusion proved by magnetic resonance angiography (MRA), who arrived at the hospital between 4.5 to 24 h after symptom onset (Table 1). The criteria for exclusion included hemorrhagic stroke; hemorrhagic transformation or recurrent stroke; myocardial infarction, atrial fibrillation and other cardiovascular diseases; treatments with thrombolytic or anticoagulants; evidence of other diseases of the central nervous system; tumor(s); the presence of recent infection; immune diseases; concomitant use of antineoplastic, immunosuppressive or immune-modulating therapies; abnormal renal or liver function; blood disorders; or diabetes mellitus. The control group consisted of 5 healthy adults matched for age, sex and vascular risk factors, including body mass index, hypertension, and hyperlipidemia.

**Table 1:** Clinical characteristics of ischemic stroke patients and vascular risk factor-matched controls.

	**IS patients (*n* = 5)**	**Controls (*n* = 5)**	***P*****-value**
Mean age (SD), yr	61 ± 11.3	59 ± 6.2	0.795
Sex, male (%)	4 (80)	4 (80)	1.000
Body mass index (BMI)	25 ± 0.8	24 ± 1.1	0.642
**MEDICAL HISTORY**			
Hypertension, *n* (%)	2 (40)	3 (60)	1.000
Hyperlipidemia, *n* (%)	4 (80)	2 (40)	0.521
Current smoking, *n* (%)	4 (80)	3 (60)	0.782
**MEDICATION, N (%)**			
Antiplatelet agent	1 (20)	2 (40)	0.660
Median NIHSS score at baseline (IQR)	12 (6–21)	0	
**OCCLUSION SITE, N (%)**			
Terminal internal carotid artery	1 (20)	0	
First segment of middle cerebral artery	3 (60)	0	
Second segment of middle cerebral artery	1 (20)	0	

*IS, ischemic stroke; NIHSS, National Institutes of Health Stroke Scale; IQR, interquartile range*.

*Hypertension was defined as systolic blood pressure ≥ 140 mmHg and/or diastolic blood pressure ≥ 90 mmHg or use of antihypertensive agents; Hyperlipidemia was defined as a total serum cholesterol level > 5.72 mmol/L and/or triglycerides >1.7 mmol/L or use of antihyperlipidemic medication*.

### Blood Collection and RNA Extraction

Blood was collected within 24 h and 7 days after symptom onset. For control samples, blood was collected only once. Blood samples were obtained in PaxGene tubes containing a reagent to stabilize RNA. Total RNA was extracted and purified from PBMCs using TRIzol reagent (Invitrogen, Grand Island, USA) according to the manufacturer's standard protocol. The concentration of RNA was determined with a NanoDrop ND-1000 spectrophotometer (NanoDrop Technologies, Wilmington, USA), and quality assessment was made by an Agilent 2100 Bioanalyzer (Agilent, Waldbronn, Germany). No samples were removed from the RNA-seq analysis due to disqualification of the blood sample or a low total RNA yield.

### Library Preparation for lncRNA Sequencing and Data Analysis

RNA extracted by TRIzol reagent with RIN>8.0 was utilized to construct a ribosomal RNA (rRNA) depletion library [VAHTSTM Total RNA-seq (H/M/R)], according to the manufacturer's recommendations. In this experiment, 15 samples from 10 individuals were used for pair-end RNA sequencing with Illumina HiSeq 4000 platform. Raw data of each samples obtained more than 10 G bases and Q30 were >95% totally. Next, clean reads were obtained by removing the adaptor sequences, reads with >5% ambiguous bases (noted as N) and low-quality reads containing more than 20 percent of bases with qualities of <20 from raw data. Then, the clean data were mapped to the human genome (GRCh38 database, NCBI) utilizing HISAT2, and HTSeq was used to calculate the gene count of mRNA and lncRNA. RNA-seq data were analyzed by Novel Bioinformatics Company (Shanghai, China).

### Prediction of Target Genes

The cis- and transregulatory effects of LncRNAs were used to target the positions of protein-coding genes and predict their functions. In the present study, protein-coding genes located within 10 kb upstream or downstream of the given LncRNAs were classified as LncRNAs of cis-regulated target genes. Furthermore, we analyzed the coexpression of LncRNAs and coding genes by calculating Pearson's correlation coefficient.

### Differential Expression Analysis

The EB-Seq algorithm was used to test the differential expression of both LncRNAs and coding genes among the groups (16). R programming was applied to analyze the lncRNA-gene pairs. The differentially expressed genes were chosen as follows: fold change >2 or <0.5; *P*-value <0.05 and false discovery rate (FDR) <0.05. Hierarchical clustering was performed based on the normalized expression of LncRNAs and mRNAs from all groups using Cluster 3.0. Heatmaps were completed with Java TreeView.

### GO and KEGG Enrichment Analysis

Gene ontology (GO) enrichment analysis and kyoto encyclopedia of genes and genomes (KEGG) pathway analysis were applied for functional annotation. The GO analysis was implemented with the UniProt and AmiGO databases (http://www.uniprot.org and http://amigo1.geneontology.org/cgi-bin/amigo/go.cgi). The KEGG database is the resource for understanding high-level functions and effects of biological systems (http://www.genome.jp/kegg/). Significance *P*-values were defined by Fisher's exact test, and FDR was calculated by the BH test. Differentially expressed genes were considered to be significantly enriched for GO and KEGG terms with *p* < 0.05.

### Coexpression Network Construction

We constructed the lncRNA-mRNA coexpression network to identify the interactions among differentially expressed LncRNAs and mRNAs (17). We calculated Pearson's correlations and chose the significant correlation between each pair of genes to construct the network. In gene coexpression networks, we chose degree centrality to determine the relative importance of a gene within a network. Degree centrality is defined as the link numbers one node has with the other. Moreover, to locate core regulatory genes, k-cores in graph theory were introduced as a method of simplifying graph topology analysis. A k-core of a protein-protein interaction network usually contained cohesive groups of proteins (18).

### Quantitative PCR Analysis

To verify the RNA-seq data, we randomly selected 6 differentially expressed LncRNAs and detected their expression changes by quantitative PCR (qPCR) (*n* = 10, 5 patients with acute ischemic stroke and 5 healthy controls). Total RNA was extracted from PBMCs with TRIzol® reagent (Invitrogen, USA) following the manufacturer's instructions. RNA quantity was determined with a NanoDrop ND-1000 spectrophotometer (NanoDrop Technologies, Wilmington, USA), and quality assessment was made by an Agilent 2100 Bioanalyzer (Agilent, Waldbronn, Germany). The total RNA was then reverse transcribed to cDNA using EasyScript First-Strand cDNA synthesis SuperMix (Transgen, Beijing, China) according to the manufacturer's instructions. SYBR Green (Roche, Basel, Switzerland) was used for qPCR. β-Actin mRNA was used as an internal control. The results were analyzed according to the 2^−ΔΔCt^ method. The 6 LncRNAs and qPCR primers are provided in Table 2. Statistical analysis was performed using Student's *t*-test, and *p* < 0.05 was considered to indicate statistical significance.

**Table 2:** List of primers for qRT-PCR.

**Name**	**Forward Primer (5^**′**^-3^**′**^)**	**Reverse Primer (5^**′**^-3^**′**^)**
β-Actin	CCAGGGCGTTATGGTAGGCA	TTCCATATCGTCCCAGTTGGT
SCARNA10	GGT CTG TAA TCT TGG TGG GCG	AGG ACC CTT GGC CCT GAT AC
TERC	CAC TGC CAC CGC GAA GAG TTG	ACT CGC TCC GTT CCT CTT CCTG
LINC01481	CGT CAC CGT CAC CTC CTG AGT AG	GGC TAG AGC AAG TTC ACA CGA CAC
FLJ23867	TGA GGA GGC TTC TGC GAG ACC	ATA CCA AGG CTG CTG GAG AGG AG
H3F3AP6	AGC TCC AGC CGA AGG AGA AGG	TCA CGG AGC GCC ACA GTA CC
TNPO1P1	TGC CTA TCT TCA GTT GCC ACA GC	ATG TTG CCT CCA AGT CCT TCA GC

## Results

### Clinical Characteristics of IS Patients

We collected 15 samples (10 ischemic stroke and 5 controls). The mean score on the National Institutes of Health Stroke Scale (NIHSS) was 12 ± 9, and the infarct volume was 42 ± 5 ml at inclusion (24 ± 0.6 h after ischemia). The mean time from stroke onset to blood collection was 24 ± 0.6 h and 7 ± 0.2 days. Baseline characteristics were generally balanced between the two groups (Table 1). Patients with acute ischemic stroke had an older age (61 vs. 59), a higher median body mass index (25 vs. 24) and hyperlipemia (4 vs. 2), although these potential differences were not statistically significant (Table 1).

### Differentially Expressed LncRNAs and mRNAs in IS Patients

Total RNA from PBMCs of all five patients (at two time points) and controls were isolated and sequenced. A total of 438 million (IS-24 h), 448 million (IS-7 d), and 433 million (control) raw reads were obtained by RNA-seq (Table 3). After quality control, approximately 415 million and 424 million clean reads were isolated from ischemic stroke patients at 24 h and 7 days, respectively, and 409 million clean reads were obtained for the control group. The sequence reads were used to map known human gene types. Approximately 86.0% (IS-24 h) and 88.9% (IS-7d) of the clean reads were uniquely mapped to the reference genome, and in the control group, approximately 91.3% of the clean reads were uniquely mapped to the reference human genome (Table 3).

**Table 3:** Summary of sequence reads mapping to genome.

**Subjects**	**Raw reads**	**Clean reads**	**Total mapped**	**Uniquely mapped**
IS 24h-1	93266260	88229464	85737538 (93.4%)	77314172 (84.2%)
IS 24h-2	84579048	80308938	79940077 (96.0%)	72561277 (87.2%)
IS 24h-3	85099148	80991756	80976131 (96.5%)	73478271 (87.5%)
IS 24h-4	82115336	77887870	78022799 (96.3%)	70670320 (87.2%)
IS 24h-5	93048884	88083076	88743881 (95.7%)	77803726 (83.9%)
IS 7d-1	86821228	81949902	81737184 (97.3%)	76647999 (91.2%)
IS 7d-2	101448746	96386482	95903673 (95.9%)	86618696 (86.6%)
IS 7d-3	82956746	77636230	77461460 (96.4%)	71018180 (88.4%)
IS 7d-4	87137944	82831000	81892575 (96.1%)	75828010 (89.0%)
IS 7d-5	90385706	85835948	84945035 (96.3%)	78942332 (89.5%)
Control-1	87788642	83192158	82064394 (96.6%)	77357888 (91.1%)
Control-2	88253988	83340744	82096397 (96.7%)	77763616 (91.6%)
Control-3	84166168	79853224	78060291 (95.7%)	73549421 (90.2%)
Control-4	83464700	78827834	77872308 (97.0%)	74052126 (92.2%)
Control-5	89547908	83945934	82706071 (96.7%)	78385062(91.6%)

*IS, ischemic stroke*.

**Figure 1:**
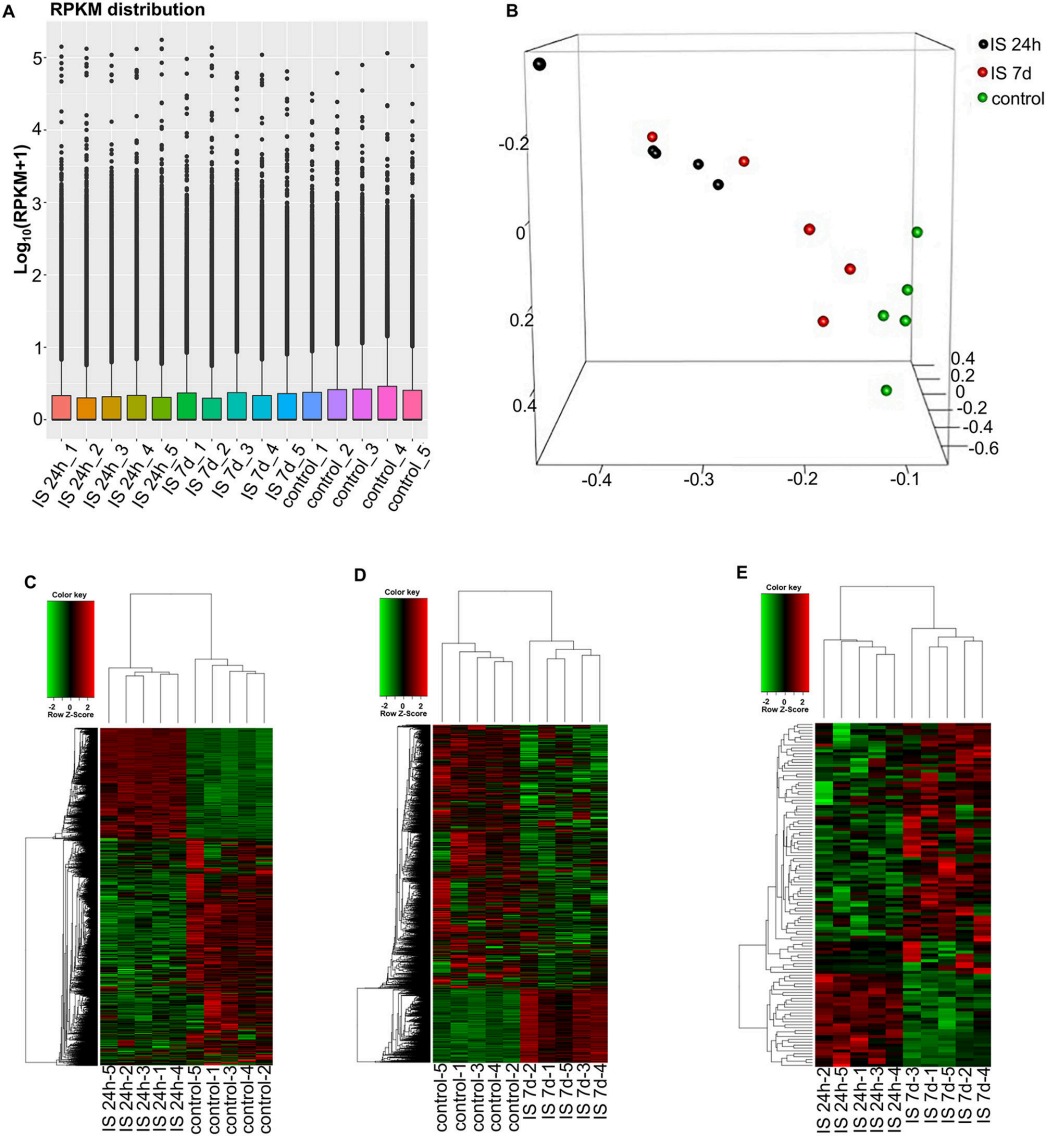
RNA-seq analysis of mRNAs and LncRNAs in IS patients. **(A)** RPKM (reads per kilobase per million) distribution for LncRNAs in all PBMCs from IS patients and healthy controls. **(B)** Three-dimensional principal component analysis (PCA) of all 15 samples to evaluate the variability of RNA-seq data. The axes represent the principal components (PC1, PC2, and PC3). **(C–E)** Cluster analysis of differentially expressed LncRNAs and mRNAs of IS patients and healthy controls. **(C)** Hierarchical clustering analysis indicated 3,009 LncRNAs and 3,982 mRNAs that were differentially expressed between IS patients at 24 h (*n* = 5) and healthy controls (*n* = 5), and the number decreased to 2,034 and 1,641 at 7 days, respectively **(D)**. **(E)** The heatmap shows that 73 LncRNAs and 36 mRNAs were differentially expressed between 24 h and 7 days in IS patients. In the color scheme, red indicates higher expression, and green indicates lower expression.

To evaluate the consistency of the sample collection and investigate the transcriptomic relationship between the 24-h and 7-day time points in the stroke patients, we performed hierarchical clustering and principal component analysis (PCA) for these samples using RNA-seq data. Both analyses can provide an overview of the similarities and dissimilarities between samples. The expression level in each sample showed no differences in terms of reads per kilobase per million (RPKM) values (Figure 1A). In the 3D-PCA plot, the PBMC samples from control subjects were grouped together (Figure 1B), which was in agreement with the hierarchical clustering analysis. In ischemic stroke patients, the PBMC samples from the two time points were grouped together with slight variation, which is acceptable considering individual differences (Figure 1B).

To investigate the key LncRNAs and mRNAs involved in ischemic stroke development, RNA-seq was performed to detect the differentially expressed LncRNAs and genes between ischemic stroke patients and the control group. The EB-Seq algorithm was used to identify the different LncRNAs and coding genes. Lastly, 3,009 LncRNAs were found to be differentially expressed, including 455 upregulated and 2,554 downregulated LncRNAs at 24 h after stroke. Moreover, there were 3,982 differentially expressed mRNAs including 1,750 upregulated and 2,232 downregulated genes. The heatmap is displayed in Figure 1C. However, the numbers of both differentially expressed LncRNAs and genes decreased at 7 days after stroke. There were 2,034 differentially expressed LncRNAs, composed of 1,859 downregulated and 175 upregulated LncRNAs. In addition, 1,641 mRNAs, consisting of 1,028 downregulated and 613 upregulated mRNAs, were detected at 7 days. The differentially expressed LncRNAs and mRNAs are demonstrated as a heatmap (Figure 1D). Comparing IS 24 h and IS 7 days, 73 differentially expressed LncRNAs (55 up- and 18 downregulated) and 36 differentially expressed mRNAs (22 up- and 14 downregulated) were identified, and the heatmap is shown in Figure 1E.

### The GO and KEGG Enrichment of Differentially Expressed mRNAs

**Figure 2:**
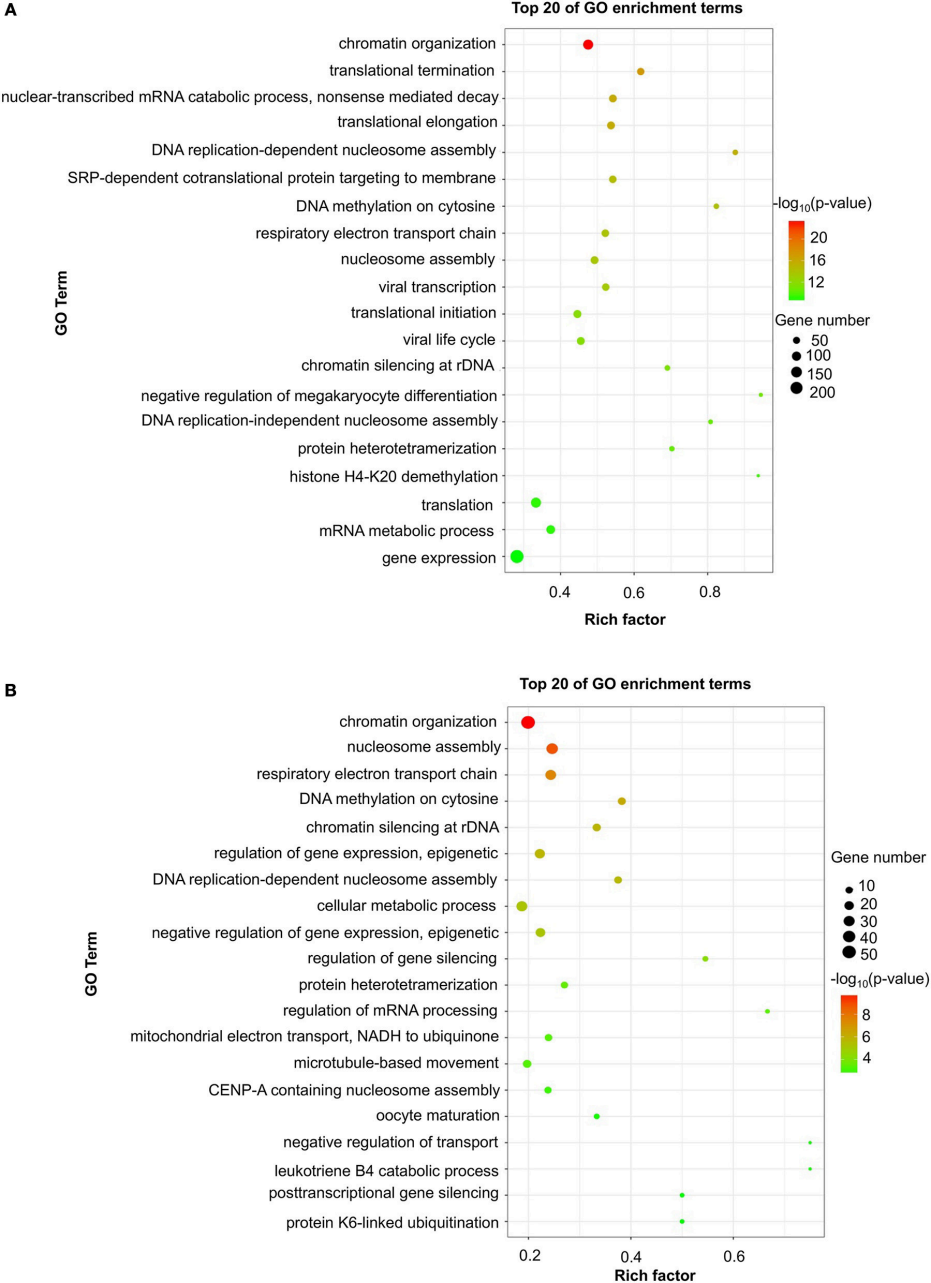
GO enrichment of differentially expressed mRNAs. Significantly enriched Gene Ontology (GO) terms of differentially expressed mRNAs in patients at 24 h **(A)** and 7 days **(B)** after stroke. The y-axis shows GO terms, and the x-axis presents counts of differentially expressed mRNAs in IS patients enriched for GO terms. The color scale represents the –log *P-*value.

To evaluate the functions of differentially expressed mRNAs, GO and KEGG pathway enrichment analyses were conducted. In the 24 h group, GO analysis was performed on 3,382 significantly dysregulated mRNAs, and the top 20 results are presented in Figure 2A. In the analysis of upregulated mRNAs, 275, 113, and 96 GO terms were significantly enriched in biological process, cell component and molecular function, respectively. In the analysis of downregulated mRNAs, 263, 66, 110 GO terms were significantly enriched in biological process, cell component and molecular function, respectively. Biological processes clustered in transcriptional and translational regulation were the most significantly enriched GO annotations at 24 h after stroke. Similarly, the top 10 results of the KEGG analysis are presented in Table 4. KEGG analysis showed that 35 pathways were included in the mRNA upregulation and that 44 pathways related to downregulated mRNAs were significantly enriched. Among these significantly enriched pathways, ribosome and lysine degradation were the most significant pathways (Table 4).

**Table 4:** Top 10 significant KEGG pathways for differentially expressed LncRNAs and mRNAs.

**Term**	**Regulation**	**Count**	***P*****-value**
**IS 24h**			
Lysine degradation	Down	17	3.40E-06
Insulin signaling pathway	Down	32	4.48E-06
FoxO signaling pathway	Down	29	2.44E-05
Thyroid hormone signaling pathway	Down	24	0.000
Adherens junction	Down	17	0.000
Renal cell carcinoma	Down	16	0.001
Osteoclast differentiation	Down	25	0.001
Neurotrophin signaling pathway	Down	24	0.002
mRNA surveillance pathway	Down	18	0.003
Prostate cancer	Down	17	0.005
Ribosome	Up	72	1.75E-36
Systemic lupus erythematosus	Up	71	3.57E-35
Alcoholism	Up	64	2.88E-20
Oxidative phosphorylation	Up	47	4.90E-15
Huntington's disease	Up	52	5.58E-12
Alzheimer's disease	Up	49	8.57E-12
Parkinson's disease	Up	42	1.84E-10
Non-alcoholic fatty liver disease	Up	38	1.15E-07
Metabolic pathways	Up	166	2.40E-06
Viral carcinogenesis	Up	43	4.85E-06
**IS 7d**			
Lysine degradation	Down	9	0.000
Inflammatory mediator regulation of TRPchannels	Down	12	0.000
Parkinson's disease	Down	14	0.001
Melanogenesis	Down	11	0.002
Phototransduction	Down	5	0.004
FoxO signaling pathway	Down	12	0.005
beta-Alanine metabolism	Down	5	0.005
GABAergic synapse	Down	9	0.007
Oxytocin signaling pathway	Down	13	0.007
mRNA surveillance pathway	Down	9	0.008
Systemic lupus erythematosus	Up	26	1.93E-13
Alcoholism	Up	26	1.51E-10
Ribosome	Up	17	1.95E-06
Alzheimer's disease	Up	19	2.98E-06
Oxidative phosphorylation	Up	14	0.000
Huntington's disease	Up	17	0.000
Viral carcinogenesis	Up	18	0.000
Parkinson's disease	Up	13	0.001
Transcriptional misregulation in cancer	Up	14	0.003
p53 signaling pathway	Up	7	0.007

*KEGG, kyoto encyclopedia of genes and genomes; IS, ischemic stroke*.

For day 7, 1,308 significantly differentially expressed mRNAs were analyzed using the GO database. The top 20 results of the GO analysis are demonstrated in Figure 2B. In the analysis of upregulated mRNAs, 215, 64, and 93 GO terms were significantly enriched in biological process, cell component and molecular function, respectively. In the analysis of downregulated mRNAs, 323, 35, and 134 GO terms were significantly enriched in biological process, cell component and molecular function, respectively. Biological processes clustered in transcriptional regulation and nucleosome assembly were the most significantly enriched GO annotations at 7 days after stroke. Moreover, the top 10 results of the KEGG analysis are shown in Table 4. KEGG analysis indicated that 19 pathways were involved in mRNA upregulation and 32 pathways related to downregulated mRNAs were significantly enriched. As expected, ribosome and lysine degradation were among the top 3 significantly enriched pathways (Table 4). However, the inflammatory mediator regulation of TRP channels and GABAergic synapses were two specifically downregulated pathways among the top 10 significantly enriched pathways compared with the 24 h group. These data suggested that inflammation might play an important role in the development of ischemic stroke.

### Venn Analysis

**Figure 3:**
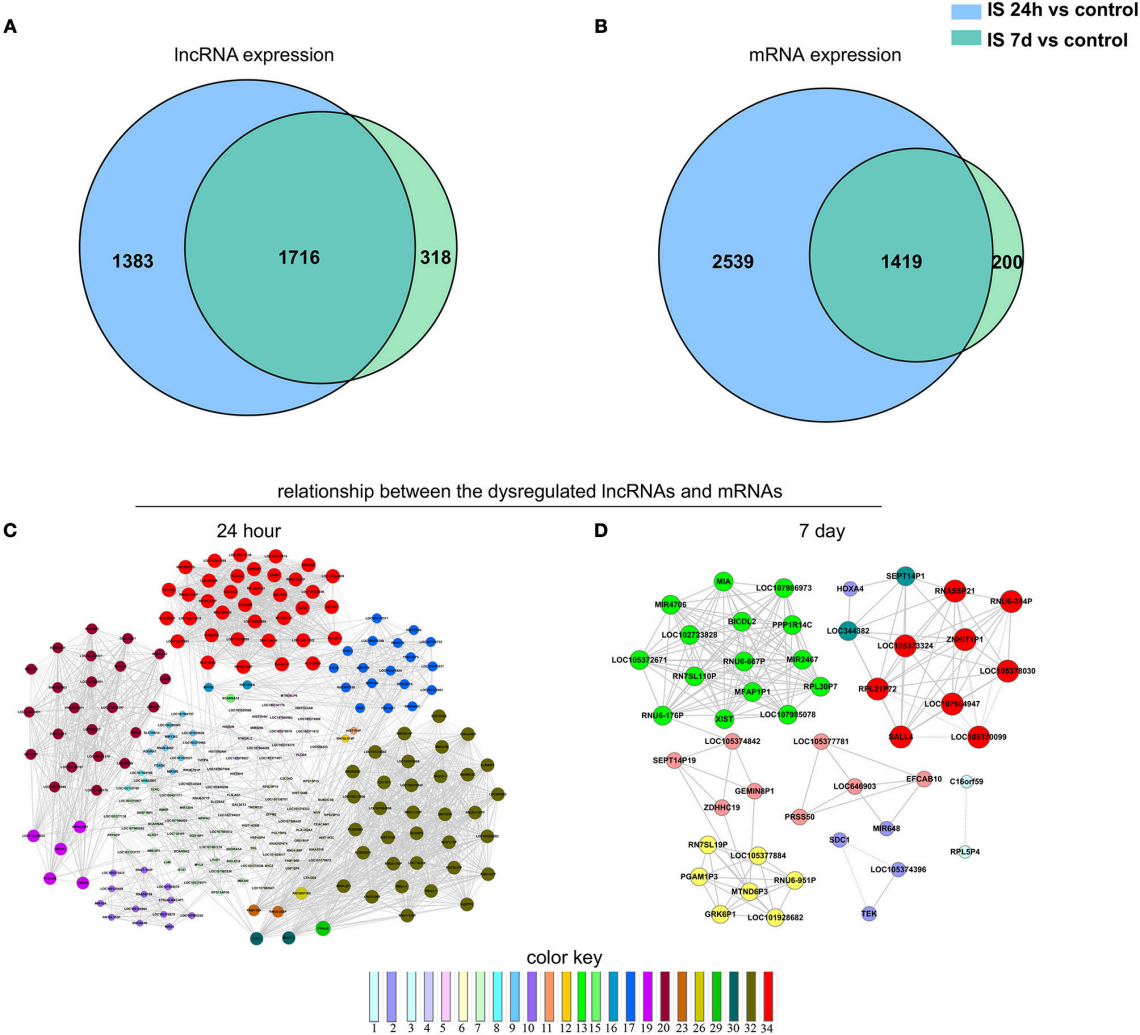
Venn analysis. **(A)** Venn diagram showing the number of specific and common LncRNAs expressed in IS 24-h patients vs. healthy controls and IS 7-day patients vs. healthy controls. **(B)** Venn diagram showing the number of specific and common mRNAs expressed in the aforementioned two comparisons. Moreover, the lncRNA-mRNA network was constructed based on the Venn analysis between the differentially expressed LncRNAs and mRNAs. **(C,D)** show the potential relationship between the dysregulated LncRNAs and mRNAs in the IS patients at 24 h and 7 days, respectively. The color key indicates differential gene degrees.

We constructed a Venn diagram to further analyze the interactions among differentially expressed LncRNAs. A total of 1,383 and 318 LncRNAs were specifically expressed in ischemic stroke patients after 24 h and 7 days, respectively. In addition, 2,539 and 200 specifically expressed mRNAs in the aforementioned comparison were used to construct a Venn diagram. As shown in Figures 3A,B, 3,135 differentially expressed genes (1,716 LncRNAs and 1,419 mRNAs) were common in the two comparisons.

To determine the potential functions of differentially expressed LncRNAs in the Venn diagrams, we predicted the possible targets of these LncRNAs in cis-regulatory relationships. We identified protein-coding genes 10 kb upstream and downstream of the differentially expressed LncRNAs. Comparing the IS 24 h patient and healthy control samples, we found 840 LncRNAs that were transcribed close to (< 10 kb) 155 protein-coding neighbors. However, only 12 cis-target protein-coding genes were found to be coexpressed with 182 LncRNAs compared between the IS 7 d patient and healthy control samples (Table 5). Pathway analysis revealed that several KEGG pathways were related only to the 24 h after ischemic stroke, such as the systemic lupus erythematosus pathway, adherent junction pathway, and tight junction pathway.

**Table 5:** Top 20 differentially expressed LncRNAs and adjacent coexpressed mRNAs.

**lncRNA**	**Expression**	**FDR**	**Protein-coding gene name**	**Regulation**
**IS 24h**				
LOC642852	Down	0	POFUT2	Down
LOC90784	Down	0	POLR1A	Down
RPL10AP8	Down	0	PTCD2	Down
FGD5-AS1	Down	0	NR2C2	Down
LOC100133091	Down	0	SPDYE16	Down
LOC107984305	Down	0	ILK	Up
LOC105377303	Down	6.25E-12	PRDM8	Down
LOC101060212	Down	8.31E-12	TBC1D3G	Down
LOC107984036	Down	1.44E-10	RAB33B	Down
LOC105373041	Down	2.19E-10	TP53BP2	Down
LOC105371449	Down	5.14E-10	PMVK	Up
CCDC58P4	Down	7.19E-10	HIGD1A	Up
RPS10P1	Down	4.25E-08	HIST1H2BF/ HIST1H4E	Up/Up
AK4P4	Down	5.36E-08	KIAA2026	Down
LOC100419838	Down	7.31E-08	ZNF320	Down
RPS10P7	Down	9.28E-08	LOC107985246	Down
LOC91450	Down	1.15E-07	TBC1D2B	Down
SNORA7B	Down	1.18E-07	EFCAB12	Down
LOC107984754	Down	2.81E-07	ANKDD1A	Down
LOC729867	Down	5.94E-07	PEA15	Up
RMRP	Up	0	SIT1	Up
SNORA59A	Up	0	VPS13D	Down
SCARNA12	Up	0	PHB2/EMG1	Up/Up
TEN1-CDK3	Up	5.55E-16	CDK3	Down
SNORD32A	Up	5.15E-13	RPL13A	Up
ILF3-AS1	Up	7.42E-12	SLC44A2	Down
SNORA5A	Up	4.09E-11	TBRG4	Up
MIR6732	Up	2.18E-10	ZC3H12A	Up
LINC01126	Up	9.07E-10	ZFP36L2	Down
SNORA7A	Up	1.97E-08	RPL32	Up
THAP7-AS1	Up	2.78E-08	THAP7	Up
LOC101929431	Up	3.32E-08	CCDC7	Up
SCARNA21	Up	1.06E-07	CHD3	Down
RASSF1-AS1	Up	2.34E-07	ZMYND10	Down
RPL24P4	Up	3.42E-06	GNMT	Down
TMEM191A	Up	1.03E-05	PI4KA	Down
EIF4EP2	Up	1.68E-05	PHB	Up
B4GALT1-AS1	Up	1.78E-05	B4GALT1	Down
FLJ38576	Up	2.43E-05	CYP4V2	Down
LOC105371038	Up	6.13E-05	MCRIP2	Up
**IS 7d**				
RNU6-384P	Down	4.06E-06	LOC107985416	Down
LOC105373836	Down	4.50E-05	CFLAR	Down
VN2R16P	Down	0.000	ZNF490	Down
LOC102724273	Down	0.000	CNOT3	Down
PMS2P9	Down	0.000	SPDYE18	Down
MIR6740	Down	0.012	ELF3	Down
KIR2DP1	Down	0.031	KIR2DL1	Down
RNU6-45P	Down	0.042	COX8A	Up
AOC4P	Down	0.047	AOC3	Down
LOC107984695	Down	0.048	LOC105370706	Down
TMPO-AS1	Up	8.03E-05	TMPO	Up
LACTB2-AS1	Up	0.015	TRAM1	Up

*lncRNA, long noncoding RNAs; FDR, false discovery rate; IS, ischemic stroke*.

### The Coexpression Network

To explore the potential interplay between LncRNAs and mRNAs, we calculated the coexpression association between the dysregulated LncRNAs and their target mRNAs based on the Venn diagrams. In total, 1,312 differentially expressed LncRNAs and 2,539 differentially expressed mRNAs were included in the coexpression network for ischemic stroke patients at 24 h and healthy controls. These differentially expressed genes constructed 3,923 network nodes and 420,593 connections comparing ischemic stroke patients at 24 h and healthy controls. However, comparing ischemic stroke patients at 7 days and healthy controls, the coexpression network profile consisted of 518 network nodes among 301 differentially expressed LncRNAs and 200 differentially expressed mRNAs with 8,185 connections (Figures 3C,D).

### Validation of the RNAs

**Figure 4:**
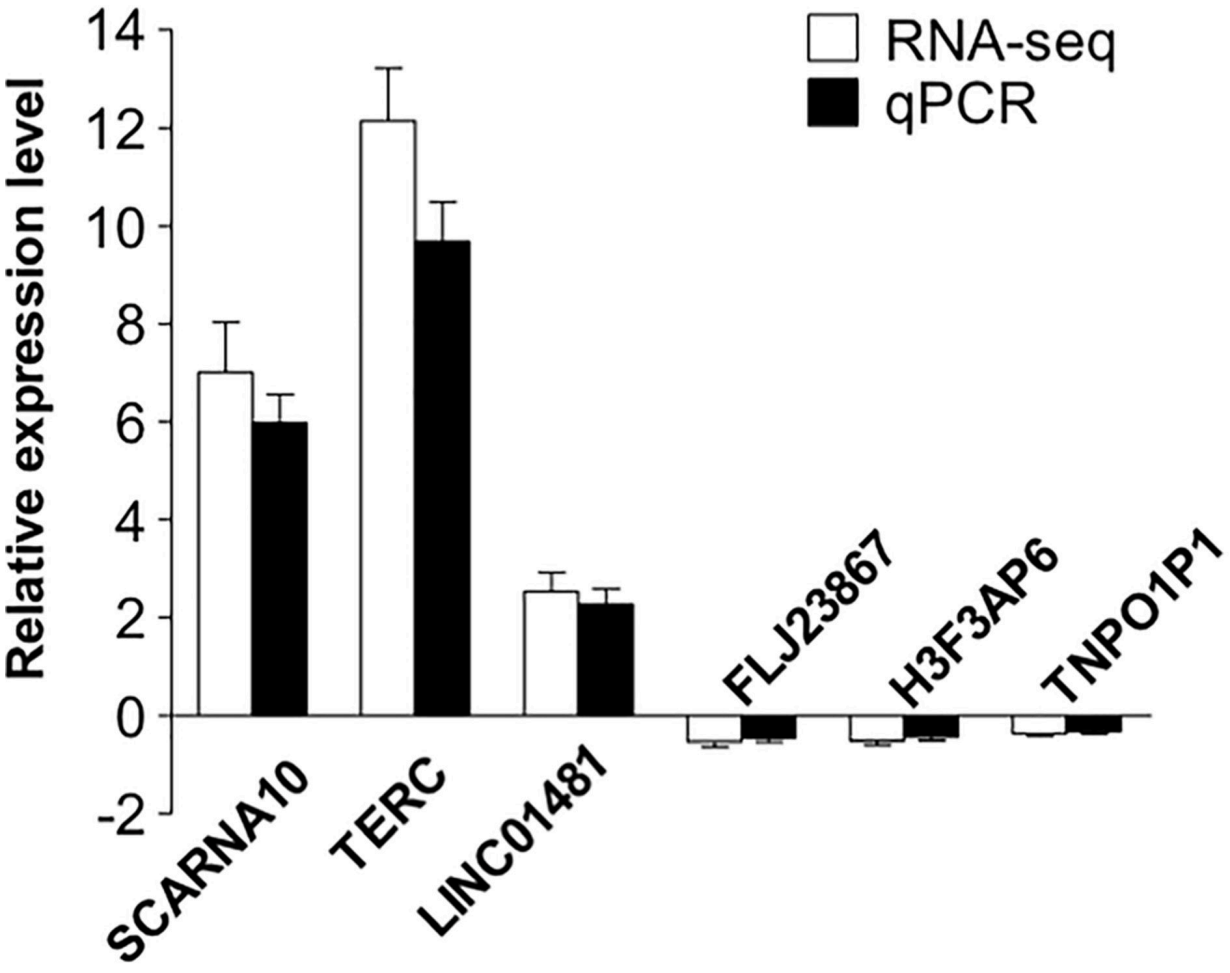
Validation of the selected LncRNAs. The verification of 6 randomly selected LncRNAs from IS patients and healthy controls by quantitative real-time PCR (qRT-PCR). Column heights represent mean fold changes in expression of the IS group. The trends in the expression of randomly selected LncRNAs detected by qRT-PCR (*p* < 0.05 vs. control) were similar to those detected by RNA-seq.

To validate our RNA-seq data independently, we randomly selected six differentially expressed LncRNAs and examined their expression patterns in ischemic stroke patients and healthy controls by qPCR. With regard to the upregulated transcripts, RNA-seq appeared to be more sensitive than qPCR. Based on the qPCR results, three LncRNAs (SCARNA10, TERC, LINC01481) exhibited higher expression (*p* < 0.01), and three LncRNAs (FLJ23867, H3F3AP6, TNPO1P1) exhibited lower expression (*p* < 0.05) in ischemic stroke patient PBMCs than in healthy control PBMCs. As shown in Figure 4, the qPCR results for all of the evaluated LncRNAs probes were concordant with the RNA-seq data. This agreement confirmed the accuracy of the RNA-seq results.

## Discussion

This is the first prospective study to demonstrate longitudinal changes in the expression of LncRNAs in human blood following acute ischemic stroke. A specific set of LncRNAs was observed to change expression over time. Notably, we found that the level of LncRNAs in the pathway of antigen processing and presentation was clearly upregulated at 24 h and had recovered to normal levels on day 7 after stroke. Moreover, inflammatory mediator regulation of TRP channels and GABAergic synapses were two specifically downregulated pathways on day 7 after stroke. These two signaling pathways are both related to inflammatory action. Their decrease may indicate the end of the inflammatory process. Our research proved the dynamic changes in the peripheral immune system after ischemic stroke at the gene level, suggesting that LncRNA might be included with mRNA and miRNA as possible biomarkers for further exploration of stroke immune system changes and causes.

Appreciation for the role of LncRNAs in the regulation of the pathophysiologic processes of ischemic stroke has increased. Although LncRNAs are usually not translated into proteins, they take part in the regulation of protein-coding genes and related signaling pathways involved in the development of multiple diseases (6). According to previous studies, aberrant expression of LncRNAs was observed in PBMCs from ischemic stroke patients using oligonucleotide microarrays (19). Through RNA-seq technology, this study demonstrated an altered gene expression profile in PBMCs during acute ischemic stroke as well. In addition, we investigated whether the expression of LncRNAs in PBMCs changed over time by serially drawing blood from every subject. Using microarray analysis, Dykstra-Aiello et al. found that 299 and 97 LncRNAs were differentially expressed in whole-blood RNA samples from male and female Canadian stroke patients, respectively, over a wide range of time points (4.4–156.0 h) (4). Similarly, by using high-throughput technologies, our study revealed that the expression levels of LncRNAs were altered in ischemic stroke patients in a Chinese Han population. Compared to microarrays, RNA-seq analysis provides a more comprehensive coverage of whole transcriptomes. As a result, we found 3,009 LncRNAs and 3,982 mRNAs that were differentially expressed in the PBMCs of patients at 24 h after stroke. On the 7th day, the number of differentially expressed LncRNAs and mRNAs decreased to 2,034 and 1,641, respectively. The expression of LncRNAs suggested an increase in stroke risk in both studies. Especially in this study, we found that the level of LncRNAs in the pathway of antigen processing and presentation was upregulated at 24 h and the level of LncRNAs in TRP and GABAergic synapses were downregulated on day 7 after stroke.

Pathway analysis showed that genes participating in the intestinal immune network for IgA production were significantly upregulated 24 h after stroke. An animal study of the intestinal flora proved that intestinal dysbiosis ameliorated ischemic brain injury through an increase in regulatory T cells and a reduction in IL-17^+^γδ T cells (20). Again, the LncRNA levels related to the pathway of the intestinal immune network for IgA production decreased to normal at day 7 in this study. Although the underlying signaling process that results in widespread immunosuppression after stroke is not well-understood, this result suggested that LncRNA may be part of the process. To provide another hope for treatment of systemic infections following immunosuppression, this result needs to be prospectively confirmed in patients with infection after stroke.

A strength of the study is the homogenous stroke population that included moderate-to-severe acute ischemic stroke subjects with anterior or middle cerebral artery occlusion presenting within 4.5–24 h of symptom onset. This study also used samples with assigned blood draw times (24 h and 7 days after stroke).

The sample size of this study was small, which may have limited our ability to identify differences between groups. In addition, evaluation of multiple genes as performed in the data analysis of RNA-seq would increase the risk of false discovery. Although correction for multiple comparisons was performed and six differentially expressed LncRNAs were validated by qPCR in this study, confirmation by an independent study is required.

In conclusion, we explored for the first time that ischemic stroke can influence the expression of LncRNAs and mRNAs in human PBMCs. GO and KEGG pathway analyses revealed that peripheral immunity may participate in the brain injury of acute ischemic stroke. Our results provide a framework for further study on the role of LncRNAs in peripheral immune system changes following ischemic stroke.

## Data Availability Statement

The datasets generated or analyzed during the current study are available from the corresponding author (Yaping Yan, Email: yaping.yan@snnu.edu.cn) on reasonable request.

## Recommended and Required Repositories

The underlying sequence data used in the study have been deposited with the Gene Expression Omnibus (GEO) under the project accession number GSE122709.

## Author Contributions

YY formulated the study concept and acquired funding for the study. LT and XY collected data. YF analyzed the data. WZ performed laboratory experiments. YY and LT wrote the manuscript. JL edited the manuscript. All authors read and approved the final manuscript.

### Conflict of Interest Statement

The authors declare that the research was conducted in the absence of any commercial or financial relationships that could be construed as a potential conflict of interest.
